# Association mapping for yield traits in *Paeonia rockii* based on SSR markers within transcription factors of comparative transcriptome

**DOI:** 10.1186/s12870-020-02449-6

**Published:** 2020-06-02

**Authors:** Na Liu, Fangyun Cheng

**Affiliations:** Peony International Institute, Beijing Advanced Innovation Center of Tree Breeding by Molecular Design, Beijing Key Laboratory of Ornamental Plants Germplasm Innovation & Molecular Breeding, National Engineering Research Center for Floriculture, Beijing Laboratory of Urban and Rural Ecological Environment, Key Laboratory of Genetics and Breeding in Forest Trees and Ornamental Plants of Ministry of Education, School of Landscape Architecture, Beijing Forestry University, Beijing, 100083 China

**Keywords:** *Paeonia rockii*, Association mapping, Yield traits, EST-SSR markers, Transcription factors

## Abstract

**Background:**

Allelic variation underlying the quantitative traits in plants is caused by the extremely complex regulation process. Tree peony originated in China is a peculiar ornamental, medicinal and oil woody plant. *Paeonia rockii*, one of tree peony species, is a precious emerging woody oil crop. However, in this valuable plant, the study of functional loci associated with yield traits has rarely been identified. Therefore, to explore the genetic architecture of 24 yield quantitative traits, the association mapping was first reported in 420 unrelated cultivated *P. rockii* individuals based on the next-generation sequencing (NGS) and single-molecule long-read sequencing (SMLRS).

**Results:**

The developed 58 pairs of polymorphic expressed sequence tag-simple sequence repeat (EST-SSR) markers from 959 candidate transcription factors (TFs) associated with yield were used for genotyping the 420 *P. rockii* accessions. We observed a high level of genetic diversity (polymorphic information content, PIC = 0.514) and low linkage disequilibrium (LD) between EST-SSRs. Moreover, four subpopulations in the association population were revealed by STRUCTURE analyses. Further, single-marker association analysis identified 141 significant associations, involving 17 quantitative traits and 41 EST-SSRs. These loci were mainly from AP2, TCP, MYB, HSF, bHLH, GATA, and B3 gene families and showed a small proportion of the phenotypic variance (3.79 to 37.45%).

**Conclusions:**

Our results summarize a valuable collection of functional loci associated with yield traits in *P. rockii*, and provide a precious resource that reveals allelic variation underlying quantitative traits in *Paeonia* and other woody oil crops.

## Background

Woody oil crops are economically essential crops globally because of their high oil content in their seeds and/or fruits, strong resistance, and stable yield. Now they have become the critical source for human edible oil, lubricants, biodiesel, cosmetics, paint and other industries [1, 2]. *P. rockii* (S. G. Haw et L. A. Lauener) T. Hong et J. J. Li belongs to the *Paeonia* section *Moutan* DC., Paeoniaceae, which is originated from northwest China and has become one of the most representative species of tree peonies. The varieties originated from the species have been cultivated widely in China as the second-largest cultivar group of Chinese tree peonies, including about 300 cultivars [3, 4]. In addition to ornamental and medicinal cultivation, *P. rockii* also presents an advantage agronomic trait for its seed yield and unsaturated fatty acid contents in seeds in recent years [5]. Therefore, *P. rockii* has been rapidly extended as an emerging woody oil plant. However, how to improve the breeding efficiency and seed yield by using the molecular breeding methods has become a top priority. The breeding system of tree peonies is predominantly outcrossing and the cultivated cultivars are of hybrid origin [3]. Conventional breeding methods for tree peony germplasm mainly include cross and selective breeding, which takes at least 10 years to develop a stable new cultivar. Therefore, the long juvenile phase and complex genetic background make it very difficult to improve the yield using traditional breeding and reverse genetics methods. The better strategies of genetic architecture in the yield traits of *P. rockii* will be required to shorten the breeding cycle and improve the production value effectively.

Currently, association mapping has been widely used in the identification and genetic testing of important quantitative trait locus (QTL) in various species [6–17]. And some progress has been made in studies on crop yield. In popular Upland cotton (*Gossypium hirsutum* L.), 172 cultivars in China and 331 polymorphic SSRs were used for association mapping of yield-related traits. Totally, 93 significantly associated loci for seven yield traits were identified across more than one environment [18]. To reveal the genetic variations of yield and yield components traits in upland cotton, 403 accessions and 560 genome-wide SSRs were used for the association mapping based on a mixed linear model. A total of 43 marker loci were detected according to the best linear unbiased prediction and in at least three of the six environments (− lg*P* > 1.30, *P* < 0.05) [19]. In *Hordeum vulgare*, a total of 379 cultivars were used for genome-wide association mapping to identify the alleles controlling yield-related traits, and 13 putative genes regulating grain traits in European cultivated barley were obtained [20]. In wheat, association mapping was performed with a mixed linear model to identify the KASP marker for yield-related traits. The results showed that Hap-5A-1/2 of TaSnRK2.9-5A was significantly associated with high thousand kernel weight, while Hap-5A-4 with high grains per spike [21]. These provide a useful reference to identify the molecular markers which are closely associated with yield traits in woody oil crops including tree peonies.

Among dozens of markers, although SNP markers are usually used in crop association mapping, SNP sites are generally biallelic polymorphisms, which are significantly lower than SSR markers, and the development and detection costs are higher. Therefore, SSR markers have become increasingly popular in association analysis because of their codominant inheritance, extensive genome coverage, chromosome-specific location, relative abundance and high throughput genotyping [22]. In particular, the SSR loci in the coding regions, as the expressed TFs, are species conserved and can directly influence the gene transcription or translation [23–25]. Currently, EST-SSRs have used for the evaluation of genetic diversity and population structure, variety identification and map construction of different species [26–33]. And EST-SSRs have been considered to be the ideal marker type for the detection of woody plants with high heterozygosity. In tree peonies, reports on the development and application of SSRs are also increasing extensively [34–40]. However, the coverage of molecular markers, especially EST-SSRs, is still limited, which limits the extensive application of functional markers in genetic diversity, population structure, LD and association analysis.

TFs can modulate gene expression and play a essential critical role in the regulation of yield. For example, IPA1 (Ideal Plant Architecture 1) can promote both yield and disease resistance by sustaining a balance between growth and immunity in rice; The CCT domain-containing gene family has significant impacts on heading date, regional adaptation and grain yield [41, 42]. In wheat, *TaNAC2-5A* overexpressing transgenic wheat lines show higher grain yield and higher nitrogen accumulation in aerial parts; A wheat CCAAT Box-binding TF can increase the wheat yield with less fertilizer input [43, 44]. In Maize, SBP-box TFs *unbranched2* and *unbranched3* can affect yield traits by regulating the rate of lateral primordia initiation; Nuclear factor Y (NF-Y) B subunits confer drought tolerance and lead to improved corn yields on water-limited acres [45, 46]. In *Brassica napus*, overexpression of the brassinosteroid biosynthetic gene *DWF4* simultaneously increases seed yield and stress tolerance [47]. In tree peonies, it is the first attempt to combine the SSR loci within TFs associated with yield to conduct the association mapping in *P. rockii*.

Taken together, the studies on the allelic variation associated with yield traits in *P. rockii* by combining association mapping and markers within TFs are rarely reported. Here, we sampled the cultivated *P. rockii* population for association mapping. After that, the genetic diversity and population structure were analyzed based on the developed EST-SSRs by comparative transcriptome. Moreover, LD and single-marker association mapping were conducted to explore the allelic effects on the natural variation of complex yield traits in the constructed population. Our results will lay a foundation to identify the linkage loci of yield traits in *P. rockii* and will be of great significance for the genetic improvement of yield traits of woody oil crops.

## Results

### Distribution and statistics of phenotypic traits in the association population

In this study, multiple methods were used to conduct descriptive statistics on phenotypic traits and determine the traits affecting the yield of *P. rockii*. The one-way ANOVA analysis by R package showed a wide range of phenotypic variation among all the 24 quantitative traits measured. The variation coefficient ranged from 12.03 to 106.63% (mean 38.60%). The average variation coefficient of branch and leaf, flower and fruit traits was 22.57, 37.21 and 49.75%, respectively (Additional file 1: Table S1–1). Correlation analysis between different traits showed 202 significant correlations (*P* < 0.05), among which 182 showed a highly significant correlation (*P* < 0.01) (Additional file 1: Table S1–2). The principal component analysis showed that the 24 quantitative traits were divided into six principal components, and the cumulative variance reached up to 73.93% (Additional file 1: Table S1–3). Then, systematic cluster analysis was carried out based on the six principal components. The results showed that the association population was divided into eight categories and 24 quantitative traits were divided into five categories where the square Euclidean distance was respectively 12 and 18 (Additional file 1: Table S1–4, Additional file 2: Figure S1).

To sum up, when individual seed fresh weight was considered as the yield index, we found that each trait was related to yield. Simultaneously, individual fruit number, individual fruit fresh weight, and individual seed number could be considered as the primary traits affecting the yield of *P. rockii*. A total of 15 traits including flower diameter, petal length, petal width, plant height, crown breadth, compound leaf length, compound leaf width, maximum basal branch angle, single fruit length, single fruit width, single fruit pericarp thickness, multiple fruit fresh weight, multiple fruit seed number, multiple fruit seed fresh weight, and multiple fruit pod fresh weight could be considered as the secondary traits. The remaining traits could be deemed to beviewed as the auxiliary reference factors.

### Genetic diversity

All the polymorphic 58 SSRs within TFs were used to evaluate the genetic diversity across 420 genotypes of cultivated *P. rockii*. Then, a total of 483 alleles were detected. The alleles per locus (*N*_A_) ranged from 3 to 16, with an average of 8. The effective number of alleles (*N*_E_) ranged from 1.02 to 7.32, with an average of 2.83. The PIC values ranged from 0.021 to 0.849 (mean 0.514) and Shannon’s information index (*I*) ranged from 0.07 to 2.18 (mean 1.13). The mean values of observed heterozygosity (*H*o) and expected heterozygosity (*H*_E_) were 0.529 and 0.561, respectively. Since *H*o was higher than *H*_E_ at 26 EST-SSR loci, the Wright’s inbreeding coefficient (*F*_IS_) ranged from − 0.828 to 0.867 with a mean value of 0.048 (Table 1). In conclusion, all the EST-SSRs showed a high level of polymorphism.

**Table 1:** Diversity information parameter at 58 EST-SSRs in the association population of *P. rockii*

Locus	***N***_**A**_	***N***_**E**_	***I***	***Ho***	***H***_**E**_	***F***_**IS**_	PIC
PS43	10	1.73	0.94	0.422	0.421	0.000	0.401
PS150	7	2.16	0.93	0.728	0.538	−0.353	0.448
PS64	8	1.97	0.94	0.507	0.491	−0.033	0.441
PS30	7	2.65	1.15	0.879	0.622	−0.412	0.562
PS113	8	2.26	0.96	0.835	0.558	−0.498	0.460
PS102	7	2.80	1.22	0.845	0.642	−0.316	0.588
PS151	13	6.06	2.04	0.506	0.835	0.394	0.816
PS8	7	2.96	1.22	0.667	0.662	−0.007	0.599
PS105	4	1.04	0.10	0.036	0.036	−0.015	0.035
PS66	14	2.89	1.38	0.338	0.655	0.483	0.613
PS160	13	4.65	1.83	0.572	0.785	0.271	0.759
PS98	6	2.14	0.89	0.552	0.533	−0.035	0.432
PS96	16	5.17	1.92	0.812	0.806	−0.007	0.784
PS75	11	2.89	1.31	0.654	0.654	0.001	0.600
PS7	10	2.98	1.47	0.439	0.665	0.339	0.635
PS131	5	2.57	1.02	0.596	0.611	0.024	0.528
PS94	7	3.40	1.42	0.544	0.706	0.229	0.664
PS25	10	2.01	1.14	0.471	0.503	0.063	0.483
PS33	12	4.05	1.60	0.766	0.753	−0.016	0.715
PS73	14	6.84	2.10	0.816	0.854	0.044	0.837
PS163	11	4.89	1.79	0.842	0.795	−0.059	0.768
PS2	5	1.37	0.51	0.288	0.271	−0.063	0.244
PS123	4	1.04	0.12	0.034	0.039	0.113	0.039
PS10	5	1.62	0.66	0.344	0.382	0.099	0.326
PS17	4	2.07	0.79	0.559	0.517	−0.082	0.405
PS19	8	3.46	1.51	0.753	0.711	−0.059	0.676
PS55	9	2.26	0.98	0.552	0.558	0.011	0.468
PS114	12	3.36	1.40	0.576	0.703	0.180	0.649
PS24	5	1.02	0.07	0.022	0.021	−0.007	0.021
PS103	10	4.08	1.61	0.810	0.755	−0.072	0.718
PS145	7	3.12	1.34	0.728	0.680	−0.070	0.632
PS21	16	7.32	2.18	0.631	0.863	0.269	0.849
PS27	5	1.46	0.59	0.274	0.314	0.128	0.284
PS59	5	1.50	0.64	0.381	0.333	−0.143	0.299
PS31	11	3.42	1.50	0.656	0.707	0.072	0.660
PS56	7	2.94	1.19	0.681	0.659	−0.033	0.595
PS57	4	1.65	0.64	0.393	0.393	0.001	0.326
PS12	8	4.57	1.71	0.778	0.781	0.004	0.753
PS47	8	2.22	0.99	0.073	0.549	0.867	0.471
PS85	6	2.51	1.14	0.600	0.602	0.003	0.550
PS93	5	2.06	0.78	0.943	0.516	−0.828	0.399
PS159	13	3.52	1.53	0.734	0.716	−0.025	0.671
PS53	4	2.31	0.94	0.593	0.566	−0.047	0.475
PS97	8	1.15	0.34	0.122	0.128	0.049	0.126
PS62	12	2.67	1.28	0.298	0.625	0.523	0.576
PS49	15	6.71	2.15	0.434	0.851	0.490	0.835
PS118	6	2.25	0.99	0.597	0.556	−0.073	0.488
PS116	5	2.24	0.97	0.646	0.553	−0.167	0.486
PS117	7	2.52	1.15	0.593	0.604	0.018	0.554
PS122	3	2.37	0.96	0.593	0.579	−0.024	0.505
PS129	6	1.88	0.81	0.167	0.469	0.644	0.397
PS142	9	2.10	1.06	0.329	0.524	0.373	0.482
PS50	9	1.74	0.88	0.353	0.425	0.169	0.389
PS91	7	2.15	0.96	0.437	0.536	0.185	0.480
PS147	16	4.13	1.68	0.834	0.758	−0.100	0.723
PS46	4	1.31	0.44	0.221	0.235	0.059	0.213
PS90	10	2.49	1.27	0.510	0.598	0.148	0.565
PS36	5	1.56	0.62	0.334	0.360	0.071	0.308
Mean	8	2.83	1.13	0.529	0.561	0.048	0.514

***N***_***A***_**Number of alleles per locus;*N***_**E**_**: Effective number of alleles;*I*: Shannon’s Information index;*PIC*Polymorphism information content;*H***_**O**_**: Observed heterozygosity;*H***_**E**_**: Expected heterozygosity;*F***_**IS**_**: Inbreeding coefficients**

### Population structure

**Fig. 1:**
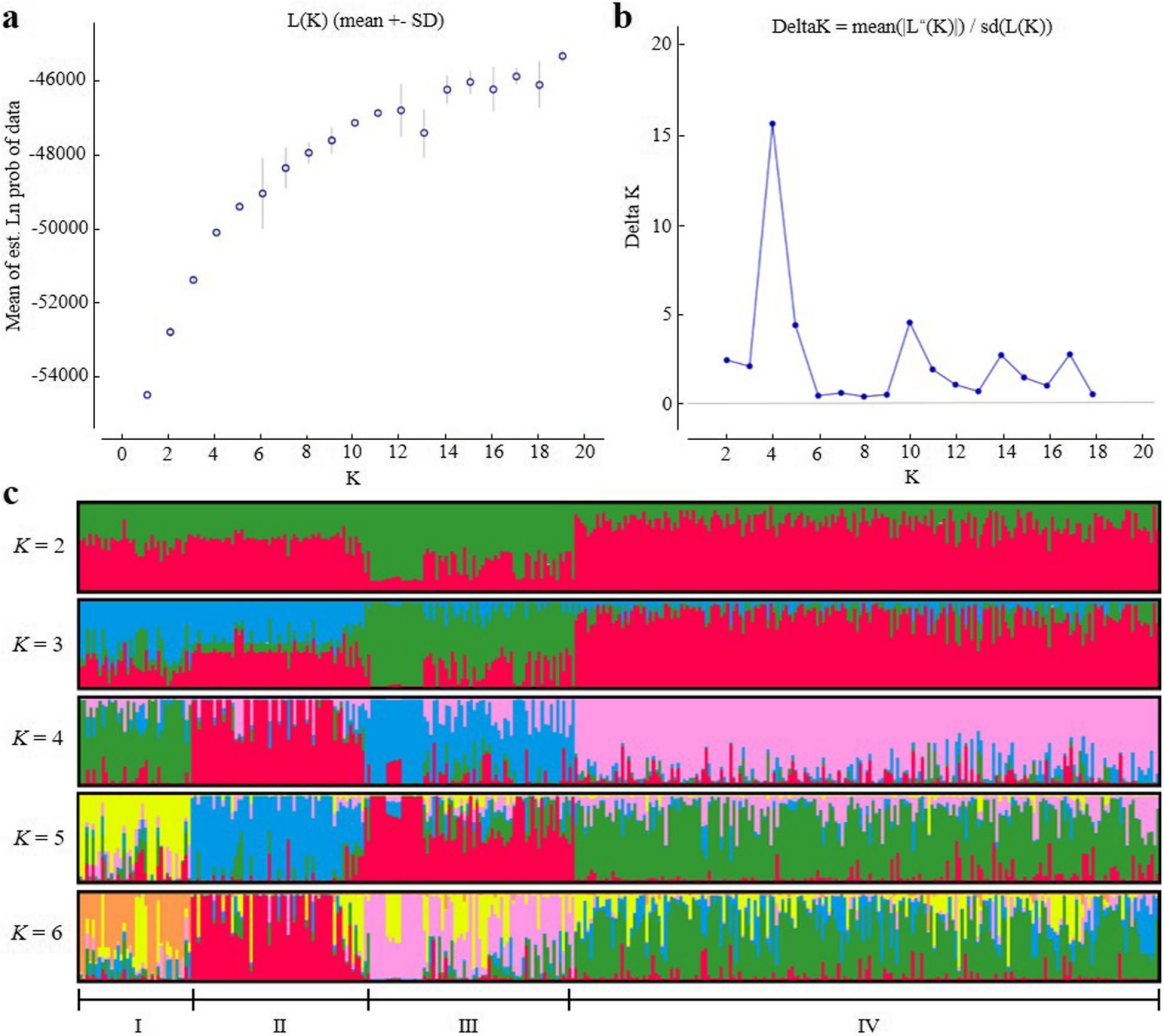
Estimation of genetic structure of 420 accessions for P. rockii population using 58 EST-SSRs based on the STRUCTURE. **a**. Log probability data [LnP (D)] for each K value (10 replicates); **b**. ΔK estimates of the posterior probability distribution of the data for a given K; **c**. Estimated population structure and clustering of the 420 P. rockii individuals with K = 2 to 6. Individuals are shown by thin vertical lines, which are divided into four major well-separated genetic clusters (I, II, III and IV) standing for the estimated membership probabilities of each individual

STRUCTURE analyses were used to determine the population structure. The result indicated that the Ln *P*(*D*) reached a mode at *K* = 4 before decreasing, and the highest delta *K* was detected when *K* = 4 (Fig. 1a, b). The species genetic structure is dicussed with the results up to *K* = 6. Bayesian methods implemented in the STRUCTURE revealed extensive admixed ancestry for each sampled of *P. rockii*. Two major well-separated genetic clusters (I-III, IV) were identified at *K* = 2–6, among which four major well-separated genetic clusters (I, II, III, and IV) were observed at *K* = 4–6 (Fig. 1c). Therefore, the 420 accessions were divided into four subpopulations.

### LD level of association population

**Fig. 2:**
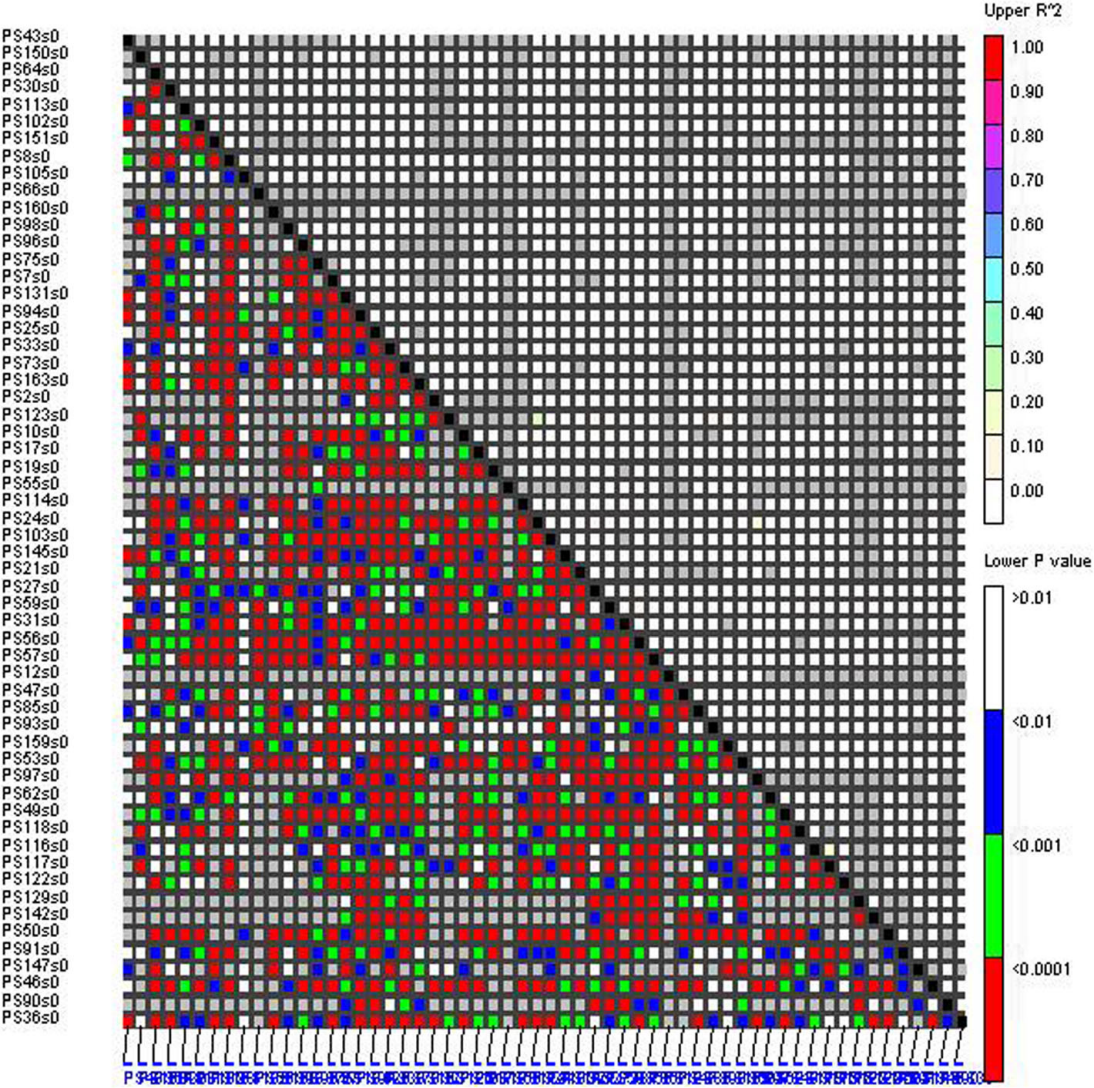
Pairwise LD (*r*^2^) between EST-SSRs. X and Y axis represent the 58 EST-SSRs. The different colors correspond to the thresholds of *r*^2^ and *P*. *r*^2^ < 0.1 and *P* < 0.001 represent linkage equilibrium, *r*^2^ > 0.1 and *P* < 0.001 represent LD

The LD of this association population was evaluated using 58 polymorphic EST-SSR markers. In a total of 1653 marker pairs (*r*^2^ ranging from 0.001 to 0.504), 64.13, 58.08 and 47.91% of EST-SSR loci demonstrated significant LD at *P* < 0.05, *P* < 0.01 and *P* < 0.001, respectively. Based on *r*^2^ estimates, only 0.67% (*r*^2^ ≥ 0.05) and 0.18% (*r*^2^ ≥ 0.1) of the loci pairs showed significant LD. Therefore, the overall level of LD between EST-SSR loci was low, and most of them were in linkage equilibrium (*r*^2^ < 0.1; *P* < 0.001), such as PS10 (PB.32979.2), PS12 (PB.59960.1) and PS17 (PB.53756.4) in the MYB gene family. Then, of the 793 assessed loci pairs (*P* < 0.001), 479 showed *r*^2^ levels > 0.005 (60.40%). Among them, several loci showed significant LD values, such as PS50 within *PSTCP11*(PB.61740.1) (Fig. 2).

### Association mapping of yield quantitative-related traits in *P. rockii*

Based on the genotype data, the *Q* matrix, the *K* matrix, and yield quantitative traits data, the MLM model was used to analyze the marker-trait associations. For floral traits associated with yield, we performed 232 (58 EST-SSRs × four traits) marker-trait association tests. Out of these, ten associations (4.31%) including 7 EST-SSR loci were significant at the *P* < 0.01 level. However, correction for multiple testing using the FDR method reduced the number to 8 (3.45%) at a significance threshold of *Q* < 0.05, involving three traits with 7 EST-SSRs. These loci explained 4.93 to 25.32% of the phenotypic variation, with an average of 11.49%. The number of associations varied across the traits was 2 (petal length) to 3 (flower diameter and petal number). One EST-SSR (PS31) was associated with at least one trait. For 3 out of the eight associations, the gene effect showed overdominance (|*d*/*a*| > 1.25). The remaining 5 associations showed additive (|*d*/*a*| < 0.50, 2) or partial to full dominance (0.50 < |*d*/*a*| < 1.25, 3) (Additional file 3: Table S2–1).

For branch and leaf traits associated with yield, we performed 464 (58 EST-SSRs × eight traits) marker-trait association tests. Among them, 102 associations (21.98%) including 39 EST-SSR loci were significant at the threshold of *P* < 0.01. However, correction for multiple testing using the FDR method reduced the number to 87 (18.75%) at a significance threshold of *Q* < 0.05, involving seven traits with 36 EST-SSRs. These loci explained 3.79 to 37.45% (mean 11.06%) of the phenotypic variation. The number of significant associations varied across the traits was 2 (crown breadth and maximum basal branch angle) to 32 (parietal lobule area), as shown in Additional file 3. Thirty-one EST-SSRs were significantly associated with at least one trait. For example, PS25 was associated with plant height, parietal lobule area, parietal lobule length, and compound leaf width. The modes of gene effect for 42 associations were overdominance, 22 were additive, and the remaining 46 were partial to full dominance (Additional file 3: Table S2–2).

For fruit traits associated with yield, we performed 696 (58 EST-SSRs × twelve traits) marker-trait association tests. Out of these, 64 associations (9.20%) including 30 EST-SSRs were significant at the threshold of *P* < 0.01. However, correction for multiple testing using the FDR method reduced the number to 46 (6.61%) at a significance threshold of *Q* < 0.05, involving seven traits with 26 EST-SSRs. These loci explained 4.45 to 30.29% (mean 11.47%) of the phenotypic variation. The number of significant associations varied across the traits was 3 (single fruit pericarp thickness, multiple fruit seed number) to 12 (single fruit number), as shown in Additional file 3. Eleven EST-SSRs showed significant associations with at least one trait. For example, PS43 was associated with single fruit number and multiple fruit pod fresh weigh. The modes of gene effect for 21 associations were overdominance, 15 were additive, and the remaining 10 were partial to full dominance (Additional file 3: Table S2–3).

### TFs associated with yield traits in *P. rockii*

Based on the results of association mapping, we observed that the floral traits were significantly associated with SSR loci within TFs from 5 gene families (AP2, NAC, GATA, HSF and GRAS), branch and leaf traits were significantly associated with 16 gene families (AP2, MYB, TCP, bHLH, HSF, GATA, B3, WRKY, etc.). Fruit traits were significantly associated with 15 gene families (AP2, HSF, MYB, WRKY, GATA and so on) (Additional file 3: Table S2). The gene families associated with all the three types of traits were AP2, GATA, HSF, and NAC, among which TFs from AP2 (29) gene family showed the highest association frequency. Moreover, all three types of traits exhibited the associations between identical traits and EST-SSRs from multiple gene families. For example, multiple fruit fresh weight was significantly associated with PS2 from the MADS-box gene family and PS30 from the AP2 gene family. Additionally, pleiotropism was generally shown in all three types of traits. For example, PS53 from the TCP gene family was significantly associated with multiple fruit seed number and multiple fruit seed fresh weight. PS12 from the MYB gene family was significantly associated with multiple fruit fresh weight, multiple fruit seed fresh weight, and multiple fruit pod fresh weight (Additional file 3: Table S2).

### SSR loci within TFs associated with fruit traits

As fruit traits were significantly associated with yield, we further dissected the results of association mapping based on the EST-SSRs associated with fruit traits. Thus, five traits with small contribution rate (single fruit number, petal number, parietal lobule area, parietal lobule length and parietal lobule width) were excluded. Further, the results showed that 49 associations were significant at the threshold of *P* < 0.01 and *Q* < 0.05, which explained 4.45 to 37.45% (mean 11.09%) of the phenotypic variance. The number of significant associations varied across EST-SSRs was 1 to 7, and 11 out of the 19 EST-SSRs exhibited significant associations with at least one trait. The modes of gene effect for 21 associations were overdominance, 15 were additive, and the remaining 13 were partial to full dominance (Table 2). PS66 (HSF), PS2 (MADS-box), PS53, PS57 (TCP), PS7 (RING), PS145 (WD), PS122, PS131 (WRKY), PS94 (YABBY), PS85 (NAC) and PS43 (bHLH) were the EST-SSRs only associated with fruit traits, so these loci could be considered as the important references for MAS breeding in *P. rockii*.

**Table 2:** Summary of significant EST-SSR marker-trait pairs from the association test results in the population of *P. rockii*

Locus	Gene family	Trait	***P-***value	***Q-***value	***R***^**2**^ (%)	d/a	2a/Sp
PS30	AP2	Multiple fruit fresh weight	0.0013	0.0224	7.66	0.642	0.513
		Single fruit length	7.51E-05	0.0027	9.38	0.632	0.715
		Multiple fruit pod fresh weight	0.0015	0.0149	7.56	3.432	0.153
		Compound leaf length	0.0005	0.0043	8.27	−0.267	0.477
		Compound leaf width	0.0007	0.0168	8.02	−0.714	0.779
PS66	HSF	Multiple fruit fresh weight	0.0007	0.0160	12.22	−0.838	0.309
		Multiple fruit seed number	0.0017	0.0241	11.54	−0.48	0.207
		Multiple fruit seed fresh weight	0.0018	0.0258	11.48	−4.877	0.058
		Multiple fruit pod fresh weight	0.0009	0.0134	11.97	−0.409	0.464
PS7	RING	Multiple fruit seed fresh weight	0.0018	0.0322	12.54	2.814	0.143
PS2	MADS-box	Multiple fruit fresh weight	0.0056	0.0498	4.45	1.193	0.332
		Single fruit pericarp thickness	0.0006	0.0142	5.73	−0.835	0.699
		Multiple fruit pod fresh weight	0.0002	0.0061	6.42	0.991	0.221
PS19	AP2	Multiple fruit fresh weight	0.0075	0.0487	12.13	−0.08	0.828
		Single fruit length	0.0032	0.0376	12.88	1.003	0.804
		Multiple fruit seed number	0.0001	0.0104	15.25	−1.517	0.539
		Multiple fruit seed fresh weight	0.0024	0.0240	13.12	−1.444	0.472
		Flower diameter	4.14E-12	1.47E-10	25.32	−0.888	1.263
PS114	GATA	Multiple fruit fresh weight	0.0022	0.0265	11.63	−1.848	0.183
		Multiple fruit pod fresh weight	0.0011	0.0134	12.15	−1.714	0.172
		Compound leaf width	0.0009	0.0111	12.29	−21.872	0.047
PS103	BZIP	Multiple fruit fresh weight	0.0019	0.0275	13.1	−18.483	0.037
		Multiple fruit pod fresh weight	0.0004	0.0080	14.23	−4.398	0.089
		Compound leaf width	0.0033	0.0236	12.66	−0.665	0.391
PS145	WD	Multiple fruit pod fresh weight	0.0067	0.0365	7.9	0.264	0.648
PS12	MYB	Multiple fruit fresh weight	0.0003	0.0106	13.41	−0.014	0.704
		Multiple fruit seed fresh weight	0.0025	0.0219	11.84	0.021	0.971
		Multiple fruit pod fresh weight	0.0002	0.0056	13.57	−0.111	0.333
		Crown breadth	0.0006	0.0420	12.92	0.121	0.732
		Compound leaf length	0.0014	0.0082	12.29	−0.406	0.608
		Compound leaf width	9.68E-27	6.87E-25	37.45	6.811	0.046
		Maximum basal branch angle	3.66E-10	2.60E-08	21.41	1.305	0.251
PS131	WRKY	Single fruit length	0.0016	0.0282	5.49	0.222	0.649
PS94	YABBY	Single fruit length	0.0029	0.0417	9.14	−0.148	0.829
PS31	AP2	Multiple fruit pod fresh weight	0.0038	0.0304	12.11	−3.884	0.102
		Flower diameter	7.43E-05	0.0018	15.04	−3.034	0.128
		Petal length	0.0011	0.0187	13.13	−0.148	0.265
		Compound leaf length	0.0005	0.0041	13.67	1.66	0.239
PS122	WRKY	Single fruit length	0.0005	0.0117	4.87	8.301	0.045
PS57	TCP	Single fruit pericarp thickness	0.0015	0.0269	4.6	−0.388	0.689
PS85	NAC	Single fruit pericarp thickness	0.0002	0.0063	9.12	5.415	0.083
PS53	TCP	Multiple fruit seed number	0.0021	0.0252	5.27	−0.798	0.306
		Multiple fruit seed fresh weight	0.0019	0.0224	5.34	−0.845	0.367
PS43	bHLH	Multiple fruit pod fresh weight	0.0094	0.0444	8.45	−0.877	0.036
PS64	HSF	Multiple fruit pod fresh weight	0.0065	0.0383	7.01	0.43	0.272
		Flower diameter	0.001	0.0173	8.3	3.048	0.215
		Compound leaf width	0.0007	0.0130	8.49	1.959	0.221
PS97	B3	Multiple fruit pod fresh weight	0.0043	0.0304	5.39	1.385	1.84
		Compound leaf width	0.0039	0.0252	5.44	−3.884	0.32

## Discussion

### Genetic diversity of the population

A vital prerequisite for dissecting population evolution and association analysis is to make clear the genetic diversity of the mapping population and the polymorphism of markers used [48]. Here, we evaluated the genetic diversity of the association population with 58 EST-SSRs developed in *P. rockii*. The results indicated that the average number of allele was 8, which was higher than that of *Ficus carica* [27], *Pistacia atlantica* [28], *Cucumis melo* [29] and cultivated *P. rockii* [39], but lower than that of wild *P. rockii* [4]. Here, we speculate that the differences should be due to the fact that the association population in this research is mainly composed of cultivated germplasm resources and there is a high level of heterozygosity in *P. rockii*. Simultaneously, the number and type of EST-SSRs and the characteristics of the test population are different.

Additionally, the population *F*_IS_ [49, 50] ranged from − 0.828 to 0.867 with an average of 0.048, of which 26 EST-SSRs were negative, indicating that there was significant heterozygous redundancy in the mapping population. We speculate that this is related to the hybrid origin and self-incompatibility of cultivated germplasm in *P. rockii* [3], which is consistent with the reported results in *Populus tomentosa* [51] and *P. rockii* [39], etc. Moreover, the positive selection of mutation loci and heterosis in the process of evolution is also critical reasons for heterozygous redundancy of *P. rockii*. Therefore, our results prove that highly heterozygous redundancy is an important biological characteristic of *P. rockii*, and further support the view that the cultivated resources in *P. rockii* are complex hybrid origin [3].

Comparing to other outcrossing woody plants, we detected a high level of genetic diversity in *P. rockii* (PIC = 0.514) [52, 53], which was higher than that of *P. tomentosa* [51] and *F. carica* [27], but lower than that in *P. atlantica* [28] and *Prunus avium* [54]. This is different from the reported results of genic-SSRs with low polymorphism level, which may be due to the hybrid origin of cultivated *P. rockii* or the fact that we sample the accessions with significant genotype differences in this study.

### Population structure

**Fig. 3:**
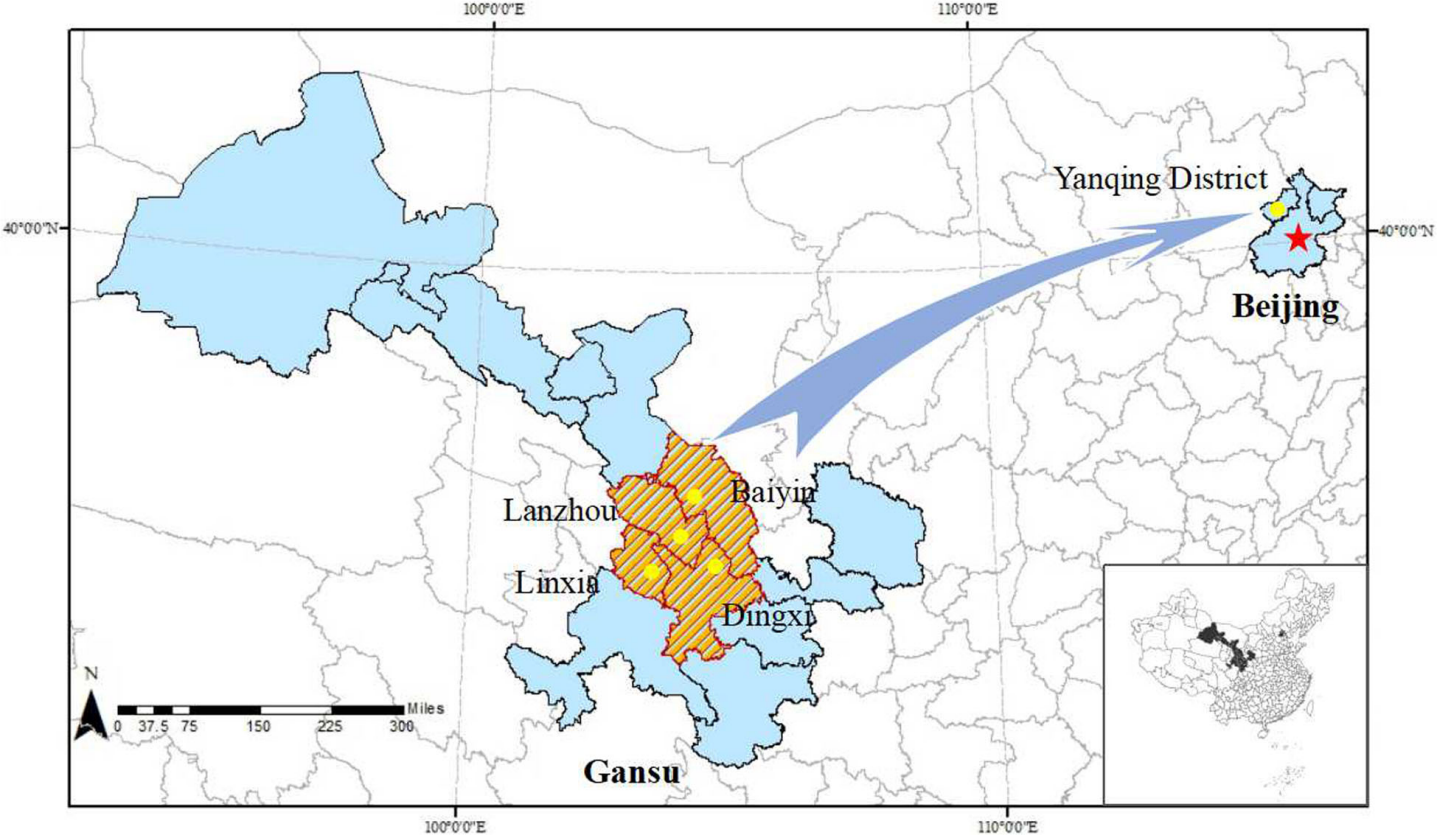
The source and distribution of population materials of P. rockii. A collection of more than 200, 000 cultivated germplasm resources of P. rockii is mainly obtained from Lanzhou, Baiyin, Linxia and Dingxi city, Gansu province in Northwest China and cultivated in open fields, using standard agronomic practices, in Beijing Guose Peony Garden of Yanqing District at Beijing, China. Figure 3 was created in ArcGIS 10.0 http://www.esrichina.com.cn

The analysis of crop population structure can not only reflect the gene exchange and affinity among individuals, but also be the premise of association mapping, which is conducive to improving the mapping efficiency and avoiding the emergence of false positives [55]. In this research, the association population was classified into four subpopulations based on STRUCTURE analyses, which is different from the previous report of three subpopulations in *P. rockii* [39]. We speculate that it is due to the differences in the EST-SSRs and population size. Moreover, the genetic information of the cultivated germplasm in *P. rockii* is mainly divided into four subgroups, which is consistent with the source of cultivated germplasm in this study, as shown in Fig. 3. We speculate that the four subpopulations may correspond to four gene pools and similarly reflect their geographic origins. It is also an essential reason for the rich genetic diversity.

### The LD level of woody plants

The analysis of LD level in population is not only the genetic underpinnings for association mapping, but also provides a reference for the selection of appropriate association analysis strategies. Generally speaking, the LD level is low in cross-pollination plants, such as *P. tomentosa* [6, 7, 56], *Eucalyptus* [57], *Gossypium hirsutum* [58] and *P. rockii* [39]. The tree peony is an outcrossing species, thus we observed a low level of LD between EST-SSRs in the association population. We speculate that a significant amount of human intervention such as cross, controlled pollination, and germplasm exchange are also essential reasons of low LD levels in *P. rockii*. Moreover, the LD level of genic-SSR loci is not representative of the whole genome or the gene interval region level, which may also lead to the low LD levels [59]. Additionally, the lack of genomic information and the unknown genetic distance of EST-SSR loci in *P. rockii* limit the analysis for the decay of LD between markers, which remains to be studied further.

### Association mapping QTL for yield quantitative traits

SSR markers based on candidate genes have higher genetic effects in regulating the expression and function of genes related to quantitative traits [60]. In this study, 41 EST-SSRs and 141 associations related to yield traits were identified in the association population of *P. rockii*. Among them, SSR loci from MADS-box, AP2, MYB, and other gene families showed high genetic effects. Simultaneously, there were not only significant associations between one trait and SSRs in multiple gene families, but also pleiotropism or co-localized associations, which was consistent with the reported results [6, 21, 39]. We speculate that these pleiotropic associations are useful for discovering the important genomic regions and valuable in trait improvement by using MAS.

In this research, we detected 176 combinations associated with three different types of traits. Still, this number was reduced to 141 after correction for multiple testing, which further improved the accuracy of the association results. Moreover, studies have shown that differences in population size and structure can cause variations in the results of association mapping. A typical association population should be a combination of multiple independent and unrelated individuals from the same region [61]. Therefore, to reduce the false positive association and provide a high precision estimation of allelic variation, we still need to verify the association results in combination with the validation populations in different regions or molecular biology experiments [62, 63].

Association mapping of complex quantitative traits in plant can detect many significantly associated markers, but explain a small portion of phenotypic variance [64]. In this study, the average interpretation rate of EST-SSRs on flower (10.81%), branch and leaf (10.40%), and fruit traits (6.53%) was low, which were consistent with the reported results [59, 61]. Besides, the analysis of genetic regulation relationship of quantitative traits in plants is helpful in utilizing the significantly association combinations for breeding. For example, the additive effect loci in the associations can account for significant genetic variation by cumulative genetic effects, while superdominant effect loci indicate that heterozygotes may superior to homozygotes. In this study, single fruit length was significantly associated with 2 EST-SSRs (PS131, PS94) with additive effects, which could add up to explain 14.63% of phenotypic variation without considering the intermarker effect. PS66 was significantly associated with the multiple fruit seed fresh weight and showed a superdominant effect (|d/a| = 4.877), indicating that individuals with PS66 heterozygous loci in the association population might generate heavier seeds. So the combination of superdominant genetic loci might show greater heterosis. All in all, the combinations of genotypes with the same positive or negative effects can be used for the early selection of target traits.

### TFs from AP2 gene family associated with yield

TFs with an APETELA2 (AP2) domain play significant roles in plant growth, development, and stress responses. Studies have also shown that TFs from the AP2 gene family are associated with crop yield improvement [65–68]. In this research, TFs from the AP2 gene family were not only significantly associated with the three types of traits, but also showed the highest association frequency. Therefore, we speculate that TFs from the AP2 gene family are important factors in regulating yield quantitative-related traits of *P. rockii*. Moreover, TFs with an AP2 domain in *Arabidopsis thaliana* and *Oryza sativa* can control seed weight and seed yield [65, 66]. In this research, PS19 (PB.50898.1) from AP2 gene family was significantly associated with seed number (multiple fruit seed number, |*d*/*a*| = 1.517) and seed weight (multiple fruit seed fresh weight, |*d*/*a*| = 1.444) in *P. rockii*, and the modes of gene effect were all overdominance (|*d*/*a*| > 1.25). Therefore, we speculate that PS19 is likely to be an essential marker for regulating the seed yield in *P. rockii*. In addition, TFs from the AP2 gene family are also significantly associated with other yield traits such as fruit size and fruit weight, which remains to be further verified in future studies.

### MYB-like TFs associated with yield

The MYB family of proteins comprise a large family of plant transcription factors, participating in the regulation of plant growth and development [69–74]. TFs with an MYB domain play an essential role in the regulation of crop yield [75–77]. In this research, in addition to the AP2 gene family, TFs from the MYB gene family also showed more associations in the mapping population. Three EST-SSRs (PS10, PS12 and PS17) from MYB gene family were significantly associated with fruit (4 associations) and branch and leaf traits (11 associations). Of these, PS12 (PB.59960.1) was significantly associated with fruit weight and seed weight-related traits, as shown in Additional file 3: Table S2. We speculate that PS12 is a key SSR loci for regulating yield of *P. rockii*. Also, a study has shown that the novel MYB-like TF *OsMPH1* can regulate plant height and improve grain yield in rice [76]. We also observed that PS10 (PB.32979.2) and PS17 (PB.53756.4) from the MYB gene family were mainly associated with branch and leaf traits. Among them, only PS17 was significantly associated with plant height in the mapping population. So we speculate that PS17 is an important SSR loci regulating plant height associated with yield in *P. rockii*.

## Conclusions

For the association mapping in *P. rockii*, we first constructed an association population consisting of 420 individuals. Then, the developed 58 polymorphic SSR loci within TFs associated with yield were selected for association mapping. Moreover, the genetic diversity and population structure were evaluated with the polymorphic loci, which proved that the population showed a high level of polymorphism and four subpopulations. Further, the results of association analysis based on single-marker showed that the 17 yield quantitative traits were regulated by 41 EST-SSR loci from 16 gene families, and 141 significant association combinations were identified. All these results furnish practical information to explore the effective functional loci associated with yield traits in tree peonies, it is also of great significance for the selection of yield-related traits and the cultivation of high-yield cultivars for oil tree peony.

## Methods

### Plant materials

More than 200, 000 seedling resources of cultivated *P. rockii* were mainly identified and collected from Gansu Province in northwest China by Beijing Guose Peony Technologies Co., Ltd. All these materials represented diverse genetic resources related to yield quantitative traits and were cultivated with general agronomic practices in the open field of Beijing Guose Peony Garden in Beijing, China (40°45′N, 115°97′E) (Fig. 3). Then based on the principle of covering the existing phenotypic variation as much as possible, we sampled a set of 420 individuals from the collection. All the evaluated samples were approximately 15 years old and covered three major flower types and eight color schemes. In total, 24 quantitative traits with stable performance were observed, demonstrating an effective assemblage of phenotypic traits.

### Phenotypic data

The 420 individuals from the association population were scored based on 24 candidate quantitative traits with at least three replicates per genotype. In total, four flower traits (flower diameter, petal length, petal width, and petal number) were measured at the full bloom using one either digital caliper (YB5001B, Kraftwelle Industrial Co. Ltd., China) or measuring tape. A total of 8 branch and leaf traits (plant height, crown breadth, parietal lobule area, parietal lobule length, parietal lobule width, compound leaf length, compound leaf width, and maximum basal branch angle) were measured. Among them, plant height, crown breadth, compound leaf length, and compound leaf width were measured using a measuring tape, the maximum basal branch angle was measured with Protractor Edge, and the other traits were measured with CI-203 laser leaf area meter (CID, USA). In addition, a total of 12 fruit traits (single fruit number, single fruit length, single fruit width, single fruit pericarp thickness, multiple fruit fresh weight, multiple fruit seed number, multiple fruit seed fresh weight, multiple fruit pod fresh weight, individual fruit number, individual fruit fresh weight, individual seed number, and individual seed fresh weight) were measured. The electronic balance was used to measure weight. The other traits were measured with the digital caliper and measuring tape (Additional file 4: Table S3).

### DNA extraction and EST-SSR markers genotyping

For each individual in association population, the total genomic DNA was extracted from silica gel-dried fresh and young leaves using the EASYspin Plus Complex Plant DNA kit (Aidlab Biotechnologies Co., Ltd. Beijing, China) according to the manufacturer’s instructions with minor modifications. Five microliter of each total DNA was assessed by 1% Tris-acetic acid-EDTA (TAE) agarose gel electrophoresis, and 1 μL was assessed using NanoDrop ND2000 [78] (A260/A280 > 1.8, 28S/18S > 1.0, DNA concentration ≥ 200 ng/μL). Then all the DNA concentrations were adjusted to 50 ng/μL for polymerase chain reaction (PCR).

We have conducted an RNA-seq experiment of flower buds of *P. rockii* ‘Jingshunfen’ and *P. rockii* ‘Fenmiantaosai’ based on NGS and SMLRS technologies [79]. The RNA-seq data have been submitted to the NCBI Sequence Read Archive (SRR9915032, https://www.ncbi.nlm.nih.gov/sra/?term=SRR9915032, and SRR10872586, https://www.ncbi.nlm.nih.gov/sra/?term=SRR10872586). Based on the sequencing data, the candidate 959 TFs from 21 gene families associated with yield based on previous reports were screened. Then a total of 166 EST-SSRs containing six nucleotide repeat types were identified, with an average of one SSR per 5.78 unigenes. Among them, 58 polymorphic EST-SSR markers have been identified and proven to be effective in *Paeonia* (Unpublished observations^1^). These 58 EST-SSRs were selected to genotype the 420 accessions (Additional file 5: Table S4).

Each PCR reaction was performed in a total reaction mixture volume of 10 μL containing 5 μL 2 × Power Taq PCR MasterMix (BioTeke, Beijing, China), 0.5 μL (10 pmol) each primer, 3.0 μL dd H_2_O and 1 μL (50 ng) template DNA. The amplification program was as follows: 5 min at 95 °C, 35 cycles of 30 s at 95 °C, 30 s at the appropriate annealing temperature, 1 min at 72 °C, and 10 min at 72 °C, 4 °C hold. The products were separated by capillary electrophoresis using an ABI3730XL capillary sequencer along with an internal size standard (Applied Biosystems, Carlsbad, CA, USA) after confirmation of PCR amplification by electrophoresis on a 1% agarose gel. The polymorphic EST-SSR loci were read with GeneMarker v1.80 software using LIZ 600 size standards (SoftGenetics, State College, Pennsylvania, USA). Subsequently, Micro-Checker v2.2.3 (http://www.microchecker.hull.ac.uk/) was applied to identify and to correct the genotyping errors [80].

### Data analysis

Descriptive statistics were performed for 24 quantitative traits, including coefficient of variation (CV/ % = standard deviation / mean × 100%), one-way ANOVA (single factor completely randomized trial design), correlation analysis, principal component analysis (PCA), and cluster analysis. All the statistics were carried out by using IBM SPSS Statistics 20.0 software and R language package.

The developed 58 polymorphic EST-SSRs were used to analyze the genetic diversity of association population. The summary statistics of *N*_A_, *N*_E_, *Ho*, *H*_E_, PIC, *I* and *F*_IS_ were calculated by GenAlEx v6.501 [81] and POPGENE v1.32 [82]. The developed 58 polymorphic EST-SSRs were also used to analyze and evaluate the population structure. The Bayesian method in the software package, STRUCTURE 2.3.4 (http://pritch.bsd.uchicago.edu/structure.html) [83], was used to infer the number of subpopulations (*K*) through an admixture model. For each value of *K* (*K* = 1–19), ten independent runs were performed with a burn-in period of 100,000 followed by 200,000 Markov Chain Monte Carlo (MCMC) replications. Then the results were submitted to the Structure Harvester (http://taylor0.biology.ucla.edu/struct_harvest/) [84]. As a result, *LnP*(*D*) and Δ*K* were used to detect the optimum *K* value [85]. Then the CLUMPP v1.1.275 was used to analyze the results from replicate analyses for optimal alignments of replicate clusters [86]. The output from CLUMPP was displayed by the cluster visualization program DISTRUCT [87]. Structural analysis was used to determine the optimum population structure and correct false positives for association mapping.

LD was measured as the squared correlation of allele frequencies *r*^2^. The *r*^2^ values between pairs of EST-SSRs (minor allele frequencies > 1%) were calculated with 10^5^ permutations using the TASSEL v2.0.1 software (http://www.maizegenetics.net/). The pairs of loci were considered to show a significant LD when *P* < 0.001. In the association mapping, the TASSEL v2.0.1 software package was used for marker-trait analysis with 10^4^ permutations by using the mixed linear model (MLM) [88]. The *Q* matrix estimating the membership coefficients for each accession was derived from the STRUCTURE runs. The relative kinship matrix (*K*) was determined by using SPAGeDi software v1.2 [89]. False discovery rate (FDR) analyses were conducted using QVALUE in R [90].

The gene effect was assessed by using the ratio of dominance (*d*) to additive (*a*) effects, which was estimated from least-square means for each genotypic class. The values of 0.50 < |*d*/*a*| < 1.25, |*d*/*a*| ≤ 0.5 and |*d*/*a*| > 1.25 were defined as partial or complete dominance, additive effects and under- or overdominance, respectively. The algorithms of dominance and additive effects were d = G_Bb_ - 0.5 (G_BB_ + G_bb_) and 2a = |G_BB_ - G_bb_|, respectively (Gij: The mean value of the phenotype corresponding to the ijth genotype; BB, bb: Different homozygous genotypes; Bb: Heterozygous genotype) [91].

## Supplementary information

**Additional file 1 Table S1.** The descriptive statistics, correlation, principal component and systematic cluster analysis of 24 quantitative traits in the association population of *P. rockii*.

**Additional file 2 Figure S1.** The clustering pedigree diagrams of 420 accessions and 24 quantitative traits in the association population of *P. rockii.*

**Additional file 3 Table S2.** Summary of significant EST-SSR marker-trait pairs from the association test results of flower, branch and leaf, and fruit traits in the association population of *P. rockii*.

**Additional file 4 Table S3.** The 24 investigation traits and measurement standard in the association population of *P. rockii*.

**Additional file 5 Table S4.** Information of 58 polymorphic EST-SSRs for association mapping.

## Data Availability

All the datasets supporting the conclusions of this article are within the paper and its Additional files. The RNA-seq data that support the findings of this study have been deposited to the NCBI Sequence Read Archive (SRR9915032, https://www.ncbi.nlm.nih.gov/sra/?term=SRR9915032 and SRR10872586, https://www.ncbi.nlm.nih.gov/sra/?term=SRR10872586). All the materials that support these findings do not contain wild resources, and all of them are cultivated germplasm resources of *P. rockii.* Beijing Guose Peony Technologies Co., Ltd. is in full compliance with institutional, national or international guidelines and has obtained appropriate permissions and business licenses.

## Abbreviations

*P. rockii*: *Paeonia rockii*
NGS: Next-generation sequencing
SMLRS: Single-molecule long-read sequencing
EST-SSR: Expressed sequence tag-simple sequence repeat
TF: Transcription factor
PIC: Polymorphic information content
LD: Linkage disequilibrium
MAS: Marker-assisted selection
QTL: Quantitative trait locus
GWAS: Genome-wide association studies
*P. tomentosa*: *Populus tomentosa*
*F. carica*: *Ficus carica*
*P. atlantica*: *Pistacia atlantica*
PCR: Polymerase chain reaction

## Footnotes

1 Liu N, Cheng FY, Guo X, Zhong Y. Development and application of microsatellite markers within transcription factors in tree peony (*Paeonia rockii*) based on next-generation and single-molecule long-read RNA-seq. Journal of Integrative Agriculture. 2020.
