# Supplementary figures and images for: Association mapping for yield traits in Paeonia rockii based on SSR markers within transcription factors of comparative transcriptome
Source: BMC Plant Biol. 2020 Jun 2;20:245. doi: 10.1186/s12870-020-02449-6 (PMC7265254; doi:10.1186/s12870-020-02449-6)

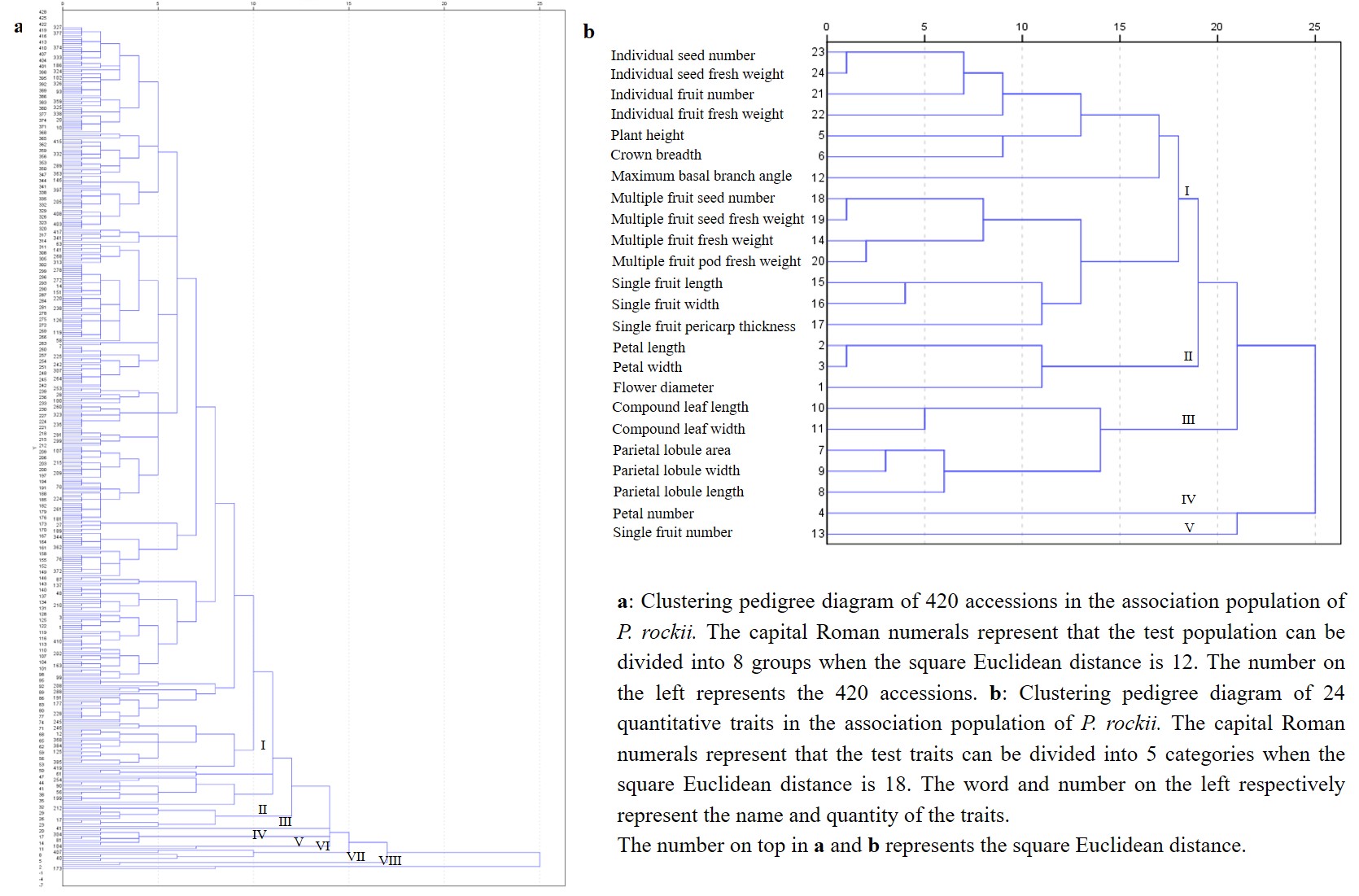

Supplement: Supplementary file 2 — Additional file 2 Figure S1. The clustering pedigree diagrams of 420 accessions and 24 quantitative traits in the association population of P. rockii. [file 12870_2020_2449_MOESM2_ESM.jpg]
